# Self-assembly of a mesoporous ZnS/mediating interface/CdS heterostructure with enhanced visible-light hydrogen-production activity and excellent stability†
†Electronic supplementary information (ESI) available. See DOI: 10.1039/c5sc01586c



**DOI:** 10.1039/c5sc01586c

**Published:** 2015-06-18

**Authors:** Kui Li, Rong Chen, Shun-Li Li, Min Han, Shuai-Lei Xie, Jian-Chun Bao, Zhi-Hui Dai, Ya-Qian Lan

**Affiliations:** a Jiangsu Key Laboratory of Biofunctional Materials , School of Chemistry and Materials Science , Nanjing Normal University , Nanjing 210023 , P. R. China . Email: yqlan@njnu.edu.cn ; Email: baojianchun@njnu.edu.cn; b State Key Laboratory of Coordination Chemistry , School of Chemistry and Chemical Engineering , Nanjing University , Nanjing 210093 , P. R. China

## Abstract

We designed and successfully fabricated a ZnS/CdS 3D mesoporous heterostructure with a mediating Zn_1–*x*_Cd_*x*_S interface that serves as a charge carrier transport channel for the first time. The H_2_-production rate and the stability of the heterostructure involving two sulfides were dramatically and simultaneously improved by the careful modification of the interface state *via* a simple post-annealing method. The sample prepared with the optimal parameters exhibited an excellent H_2_-production rate of 106.5 mmol h^–1^ g^–1^ under visible light, which was 152 and 966 times higher than CdS prepared using ethylenediamine and deionized water as the solvent, respectively. This excellent H_2_-production rate corresponded to the highest value among the CdS-based photocatalysts. Moreover, this heterostructure showed excellent photocatalytic stability over 60 h.

## Abstract 2

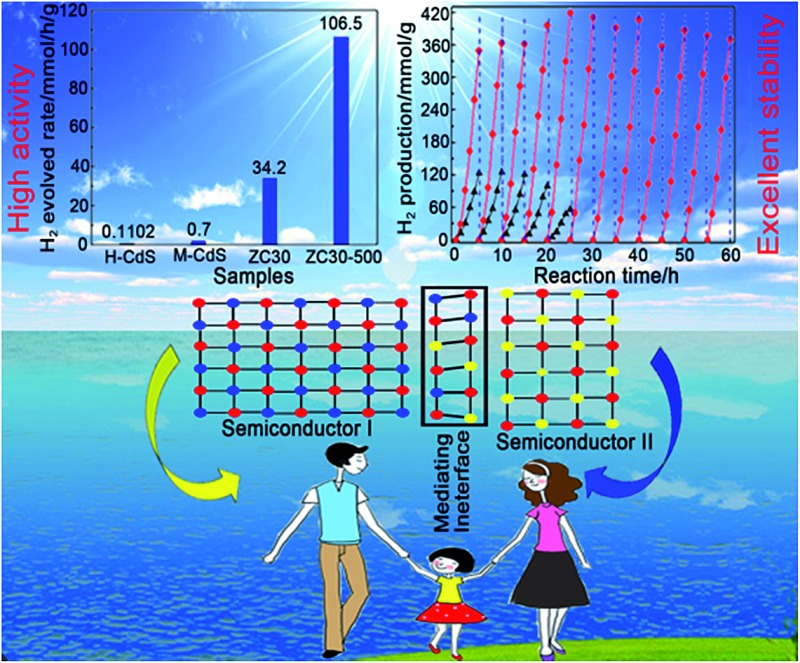
We designed and successfully fabricated a ZnS/CdS 3D mesoporous heterostructure with a mediating Zn_1–*x*_Cd_*x*_S interface.

## Introduction

Directly transforming solar energy into clean and renewable hydrogen *via* water splitting while using suitable photocatalysts is especially important for resolving the ever-increasing energy crisis. Various nanomaterials,^1^ particularly semiconductors,^2^ have been investigated as photocatalytic hydrogen-evolution catalysts since TiO_2_ was first reported to split water into hydrogen.^3^ Metal sulfide semiconductors exhibit a suitable bandgap (*E*_g_) that corresponds to visible light (approximately 44% of solar energy);^4^ they also exhibit excellent photocatalytic activity.^2c,5^ Among these sulfide semiconductors, CdS has been extensively investigated for hydrogen production because of its suitable *E*_g_ which is approximately 2.4 eV and its very negative conduction band (CB).^2a,5d,6^ However, the fatal drawbacks of CdS, which include (i) a low H_2_-evolution rate and (ii) a very limited lifetime arising from the rapid recombination of photo-excited charge carriers and the oxidization of CdS, respectively,^5d,6a^ restrict its practical application. As an effective solution to the low H_2_-evolution activity, constructing CdS-based heterojunctions, such as TiO_2_/CdS, ZnO/CdS, g-C_3_N_4_/CdS and ZnS/CdS,^5d,7^ has become a widely adopted strategy for improving the spatial separation of photo-generated electron/hole pairs. Nevertheless, the interface of the semiconductor heterojunction, which is an important factor that affects photocatalytic activity, is rarely investigated. Modulation of the matching of the lattice constant and the band structure in heterojunctions is difficult because of the limited number of candidate semiconductors with an appropriate intrinsic lattice constant and band structure.^8^ Consequently, the resulting semiconductor heterojunction may have problems involving large lattice stress, high defect concentration and poor band-structure matching, which may inhibit the improvement of photocatalytic activity.^9^ Inserting a conductive layer (such as Pt) that serves as a transport channel can dramatically improve the interface conductivity.^5b,8,10^ However, the complicated process, poor ability to mediate the interfacial state, and the high cost of noble metals restrict its application.

With respect to the limited photocatalytic stability of CdS, passivation layers and surface modifications have been adopted to protect it from oxidation.^7a,11^ Both of these methods can increase the lifetime of CdS but at the cost of deteriorating its H_2_-production rate, which is because of the inhibition of photocatalytic reactions by the passivation layer and the contamination of the heterostructure.^5d,7a^ Indeed, the low H_2_-production activity and limited photocatalytic stability primarily result from the poor separation and transport efficiency of the charge carriers. If we can embed a modulating interface with a tunable lattice constant as well as band structure, and enhance the interface state between the semiconductor heterostructure to modify the charge separation and transport time and efficiency,^12^ the H_2_-evolution rate and photocatalytic stability could be effectively improved simultaneously.^5d,13^ Therefore, developing a semiconductor heterostructure with high photocatalytic activity and stability *via* carefully mediating the interface state is a subject of especial importance from both scientific and practical perspectives.

**Scheme 1:**
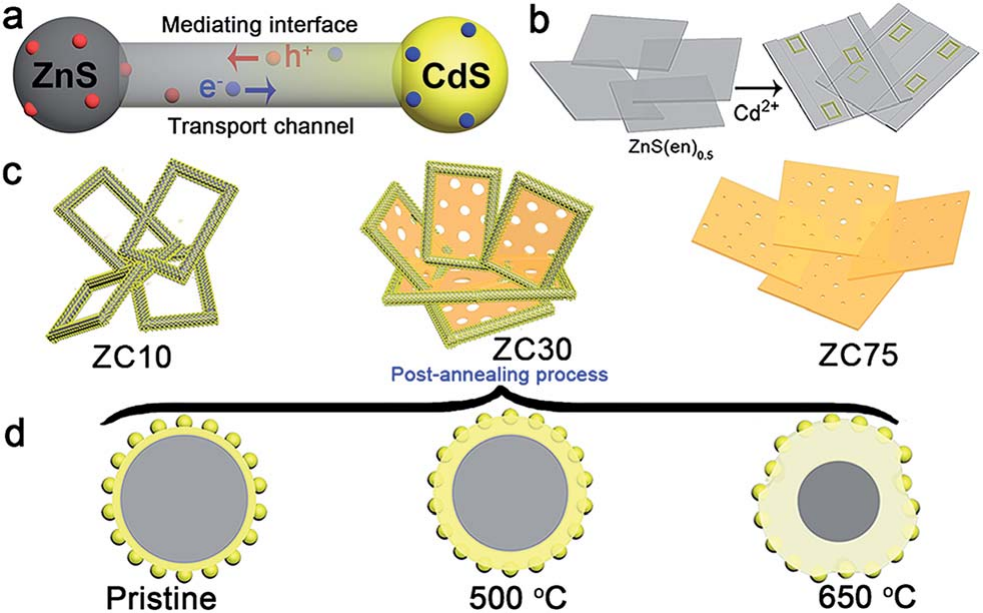
(a) Scheme of the mechanism for improving charge-carrier transport in a ZnS/Zn_1–*x*_Cd_*x*_S/CdS heterojunction. (b) Schematic illustrating the structural evolution of the mesoporous nanoframe derived from ZnS(en)_0.5_ nanosheets. (c) Morphology of ZC*X* samples with different Cd^2+^ content. (d) Effect of the post-annealing process on the microstructure of ZC30.

In view of the aforementioned ideas, a ZnS/CdS porous heterostructure with a mediating interfacial layer acting as a transport channel (Scheme 1a) has been prepared using cation exchange^14^ and post-annealing methods on the basis of the following points: (i) ZnS/CdS exhibits quasi-type II characteristics and efficient photocatalytic hydrogen evolution because of the acceptor states (I_s_, V_Zn_) in ZnS.^5*d*^ Moreover, the extensively investigated Zn_1–*x*_Cd_*x*_S with tunable parameters and lower acceptor states than those in ZnS was selected as an interfacial layer;^15^ (ii) ZnS-ethylenediamine (ZnS(en)_0.5_) was adopted as a template for fabricating the porous ZnS/Zn_1–*x*_Cd_*x*_S/CdS heterojunction,^16^ which is especially important for the water-splitting reaction because of its surface reaction characteristics;^5a,c,17^ (iii) a post-annealing process, which has been widely employed to improve the properties of oxides but has rarely been used in sulfide heterojunctions,^16a,18^ was adopted to improve the interface state and photocatalytic activity of the heterostructure sample whilst maintaining its heterojunction.

Herein, a ZnS/Zn_1–*x*_Cd_*x*_S/CdS three-dimensional (3D) mesoporous heterostructure that exhibited a very high charge separation efficiency and H_2_-evolution rate was designed and successfully fabricated for the first time. The mediating Zn_1–*x*_Cd_*x*_S interface, which possessed a tunable lattice constant and band structure, acted as the charge-carrier transport channel, which favored the improvement of the charge separation efficiency. Moreover, the H_2_-production rate and the stability of the heterostructure involving two sulfides were dramatically improved simultaneously *via* the careful modification of the interface state *via* a simple post-annealing method. The sample prepared with the optimal parameters exhibited an excellent H_2_-production rate of 106.5 mmol h^–1^ g^–1^ under visible light, which was considerably greater than that of the other previously reported CdS-based photocatalysts. Moreover, the heterostructure exhibited excellent photocatalytic stability over a period of 60 h. The well-modulated interface state is responsible for the substantially improved photocatalytic activity and stability.

## Results and discussion

**Fig. 1:**
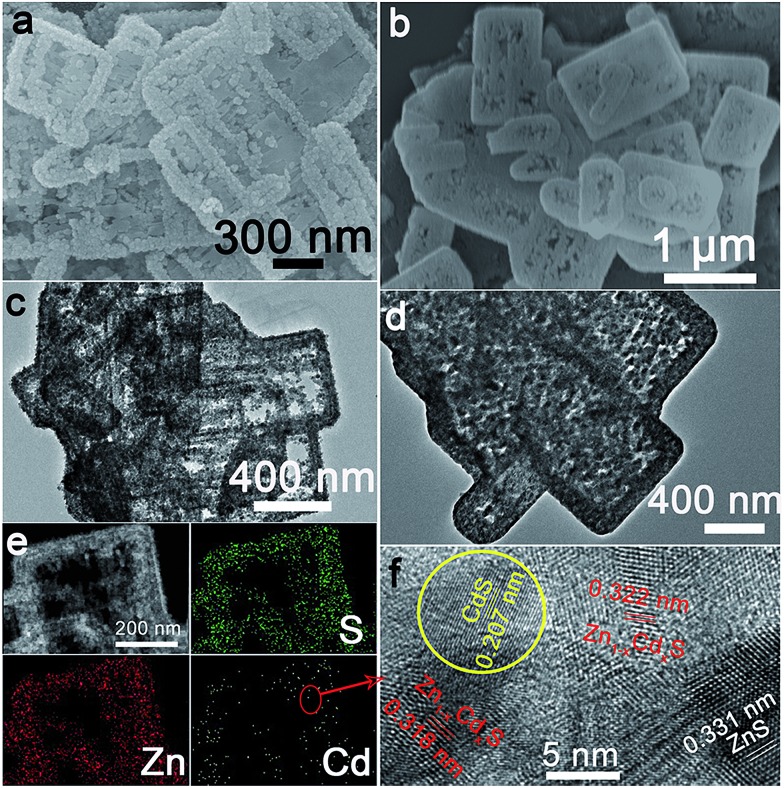
Scanning electron microscopy (SEM) and transmission electron microscope (TEM) images of (a), (c) ZC30 and (b), (d) ZC30-500, respectively. (e) Element mapping and (f) HRTEM image of ZC30.

ZnS(en)_0.5_ nanosheets, which were synthesized using a modified solvothermal method,^2*c*^ were reacted with different concentrations of Cd^2+^ at 140 °C. A series of samples with nominal Cd/Zn molar ratio *X* were labeled as ZC*X* (*X* = 0, 2, 6, 10, 30, 40, 75 at%). Scheme 1b shows the morphology evolution of the ZnS/Zn_1–*x*_Cd_*x*_S/CdS nanoframe with increasing Cd/Zn ratio. The ZnS(en)_0.5_ nanosheets were transformed into disordered porous ZnS with the gradual dissolution of EDA molecules (Fig. S1†), which then reacted with Cd^2+^ and formed the regular rectangular nanoframe ZnS/Zn_1–*x*_Cd_*x*_S/CdS heterostructure. As confirmed by the time dependence of the microstructure (Fig. S2†), Cd^2+^ sculpted the ZnS(en)_0.5_ nanosheets layer-by-layer and the rectangular nanoframe morphology was finally synthesized *via* a self-assembly process (Scheme 1c and Fig. S3†) because of the larger size of the ZnS(en)_0.5_ nanosheets compared to the reaction-zone width.^19^ As shown in Scheme 1c, the rectangular nanoframe was transformed into nanosheets with the Cd^2+^ content increasing from 10 to 75 at% (Fig. S3†), with ZC30 possessing a 3D porous microstructure constructed by nanoframes connected with porous nanosheets (Fig. 1a and c). To further improve its interface state, ZC30 was post-annealed at different temperatures; the resulting samples were abbreviated as ZC30-*Y* (*Y* = 450, 500, 550, 650 °C). Scheme 1d shows the cross-section microstructural evolution of ZC30 at different post-annealing temperatures. The post-annealing process at higher temperatures may have facilitated the reaction between ZnS, CdS and the Zn_1–*x*_Cd_*x*_S interfacial layer, and decreased the Cd^2+^ concentration in the interface solid solution and the size of the CdS quantum dots (CdS QDs). ZC30-500 exhibited a similar microstructure, although it is less porous when compared to pristine ZC30 (Fig. 1b and d); in contrast, ZC30-650 presented a denser and destructed microstructure (Fig. S3f†). As shown in the energy-dispersive X-ray (EDX) spectrum (Fig. S4†), the ratio of Cd/(Zn + Cd) in the ZC10 and ZC30 samples from the EDX results was very near to the setting value, which indicated that Cd^2+^ reacted with sufficient ZnS *via* a cation exchange reaction. However, the samples with a larger Cd^2+^ content showed a much smaller ratio of Cd/(Zn + Cd) than the setting value, which may have been attributed to the inhibited reaction between ZnS and Cd^2+^ by the interfacial layer. Especially, for the heterostructure sample with a Cd/Zn ratio of 150 at%, the Cd/(Zn + Cd) ratio from the EDX results (79.2 at%) did not increase dramatically in comparison with ZC95. Moreover, no remarkable composition variation was observed in the post-annealed samples (Fig. S5†).

The X-ray diffraction (XRD) patterns of the samples with incremental Cd^2+^ content (Fig. S6a†) showed an increase of hexagonal and cubic phases of CdS resulting from the limited S source.^20^ However, the characteristic peak of ZnS did not disappear even in ZC75, further confirming that the interfacial solid solution inhibited complete cation exchange between ZnS and Cd^2+^. Moreover, the peak corresponding to Zn_1–*x*_Cd_*x*_S slightly shifted to lower 2-theta values with increased Cd^2+^ content, and ZC30 exhibited a similar phase structure but with shifted peak positions compared to those of a ZnS and CdS mechanical mixture (Fig. S7†), thereby indicating the formation of a Zn_1–*x*_Cd_*x*_S phase. As shown in Fig. S6b,† the post-annealing process dramatically improved the crystallinity of ZC30 without the formation of any extra phases. The peak corresponding to Zn_1–*x*_Cd_*x*_S shifted to higher 2-theta values with increasing temperature, indicating that the content of Cd in Zn_1–*x*_Cd_*x*_S decreased because of the reaction between ZnS and the interfacial layer at high temperatures. Fortunately, the ZnS/Zn_1–*x*_Cd_*x*_S/CdS heterostructure was well maintained even at 650 °C because of the presence of the interface.

To further confirm the ZnS/Zn_1–*x*_Cd_*x*_S/CdS heterostructure in the as-prepared samples, element mapping and high-resolution TEM (HRTEM) were used to investigate the element distribution and phase structure of ZC30 (Fig. 1e and f). S and Zn were distributed evenly throughout the entire skeleton of the nanoframe, whereas Cd was primarily distributed at the edge of the nanoframe; this result was further confirmed by the smaller ratio of Cd/Zn at the center of the nanoframe (Fig. S8†). As shown in Fig. 1f, the lattice fringe of the nanoregion centralized at the edge of the nanoframe could be indexed as CdS. The lattice constant of Zn_1–*x*_Cd_*x*_S surrounding the CdS QDs increased as the distance to the CdS nanoregion increased, and the lattice fringe of ZnS was observed far from the CdS nanoregion. This composition gradient (corresponding to different band structures) and the *in situ* formation of a stably embedded interface could reduce heterostructure stress and defect concentration and hence improve the separation and transport efficiency of the charge carriers.^9a,b^ Compared with the pristine ZC30, the ZC30-500 possessed a higher crystallinity with smaller CdS QDs. Fortunately, the CdS QDs could still be observed in ZC30-650 (Fig. S9†), indicating the important role of the interface in maintaining the ZnS/Zn_1–*x*_Cd_*x*_S/CdS heterostructure.

**Fig. 2:**
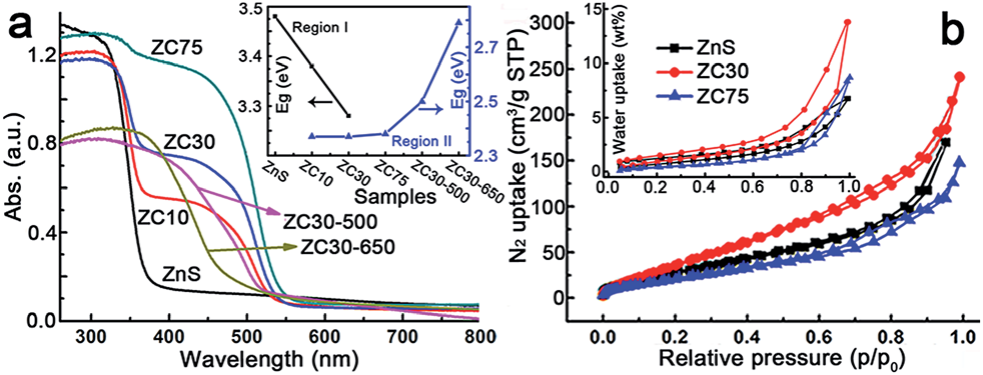
(a) Effects of Cd content and the post-annealing process on the UV-visible diffuse reflection spectra and band gaps (inset) of the heterostructure samples. (b) Nitrogen and water vapor (inset) adsorption/desorption isotherms of the heterostructure samples.

The effects of the Cd^2+^ concentration and post-annealing process on the band structure of the ZnS/Zn_1–*x*_Cd_*x*_S/CdS heterostructure were investigated using UV-visible diffuse reflection spectroscopy and are exhibited in Fig. 2a. The band edge of the heterostructure dramatically shifted to longer wavelengths in the sample with only 10 at% Cd^2+^. All of the samples exhibited similar *E*_g_ irrespective of the Cd^2+^ loading, which was in complete contrast to the *E*_g_ values of the Zn_1–*x*_Cd_*x*_S solid solution.^12*a*^ This result may have been attributed to the formation of CdS QDs resulting from the cation-exchange characteristics and the larger radius of Cd^2+^ (0.97 Å) relative to that of Zn^2+^ (0.74 Å).^14a,21^ Notably, the spectra of ZC10 and ZC30 showed two band edges, and the entire diffuse reflection spectra were divided into two regions, with regions I and II corresponding to absorption in the UV and visible regions, respectively. *E*_g_s in the visible and UV regions were calculated by the Kubelka–Munk (KM) method (Fig. S10†) and shown in the inset of Fig. 2a. The *E*_g_ in region I, which represented the Zn_1–*x*_Cd_*x*_S solid solution, decreased with increasing Cd^2+^ concentration, which was consistent with the XRD results. The *E*_g_s in region II didn't change dramatically for the samples ranging from ZC10 to ZC75. ZC30-500 showed a slightly larger *E*_g_ of CdS (2.50 eV) than that in ZC30 (2.37 eV). This can be explained by the decreased size of the CdS QDs (Fig. S9a†) resulting from the reaction between a small amount of CdS and the interfacial layer.^21^ When further increasing the temperature to 650 °C, the drastic reaction of Zn_1–*x*_Cd_*x*_S in ZC30-650 as confirmed by the XRD patterns (Fig. S6†) dramatically increased its band gap from 2.37 to 2.79 eV. The nitrogen and water vapor adsorption/desorption isotherms of the photocatalysts with different Cd concentrations are presented in Fig. 2b. ZC30 exhibited the highest water vapor and N_2_ adsorption volumes and a specific surface area (*S*_BET_) of 105 m^2^ g^–1^ (Fig. S11†), which was much larger than the other photocatalysts.^2c,15,22^ Fortunately, ZC30-500 exhibited a relatively high *S*_BET_ of 43.6 m^2^ g^–1^, whereas ZC30-650 showed a very low *S*_BET_ of 14.5 m^2^ g^–1^ (Fig. S12†).

**Fig. 3:**
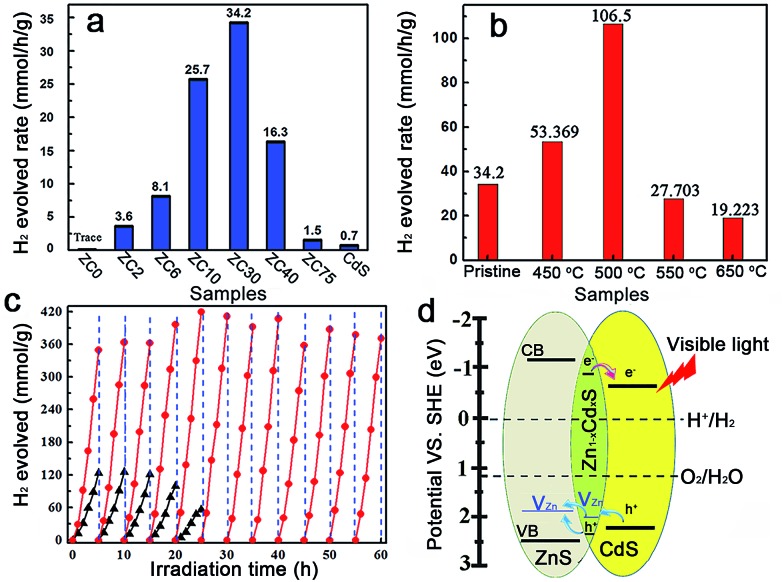
(a) Photocatalytic H_2_-production activities of the ZC*X* samples and (b) ZC30 post-annealed at different temperatures in a 0.35 M Na_2_S and 0.25 M Na_2_SO_3_ mixed aqueous solution under visible-light irradiation. (c) Comparison of the photocatalytic stabilities of pristine ZC30 (triangle) and ZC30-500 (circle). (d) Schematic illustrating charge transfer and separation in ZnS/Zn_1–*x*_Cd_*x*_S/CdS.

The photocatalytic production of H_2_ by ZnS/Cd_*x*_Zn_1–*x*_S/CdS heterostructures with different Cd^2+^ content was compared under visible-light irradiation (*λ* ≥ 420 nm) (Fig. 3a).^2*c*^ In contrast to the negligible visible-light hydrogen evolution activity in mesoporous ZnS, the H_2_-production rate drastically increased with the content of Cd^2+^ increasing from 2 to 30 at%. Further increasing of the amount of Cd led to a reduction in the H_2_-production activity. ZC30 exhibited the highest H_2_-evolution rate of 34.2 mmol h^–1^ g^–1^. The interface with a suitable band structure and lattice constant in ZC30 acted as a transport channel and increased the photocatalytic activity, whereas excess CdS may have acted as charge recombination centers and decreased the photocatalytic activity. Moreover, the maximum *S*_BET_ and water vapor adsorption of ZC30 increased the number of reaction sites and the absorption capacity for photons and water molecules. The charge-carrier separation and transport efficiencies were further confirmed *via* electrochemical impedance spectroscopy (EIS) (Fig. S13†). The spectrum of ZC30 showed a smaller semicircle in the middle-frequency region when compared to ZC75 and CdS, indicating that it possessed the fastest interfacial electron transfer arising from the well-modulated interfacial layer. To qualitatively confirm the extremely important role of the interfacial layer on the H_2_-production activity, a sulfur source (Na_2_S) was added with the Cd^2+^ to inhibit the formation of the Zn_1–*x*_Cd_*x*_S interface layer.^5d,23^ As shown in Fig. S14,† both the intensity of the CdS and ZnS phase got stronger with an increasing amount of the sulfur source. Correspondingly, the H_2_-production activity decreased dramatically because of the inhibited formation of Zn_1–*x*_Cd_*x*_S.

The post-annealing method, which has been extensively employed to improve the properties of oxides but has rarely been applied to sulfide heterojunctions,^16a,19^ was adopted to investigate the effects of interfacial states on the photocatalytic H_2_-evolution activity of ZC30. As shown in Fig. 3b, the photocatalytic H_2_-evolution activity first increased and then decreased with increasing post-annealing temperatures. ZC30-500 exhibited the maximum H_2_-evolution rate of 106.5 mmol h^–1^ g^–1^, which was 152 and 966 times greater than those of CdS prepared using ethylenediamine and deionized water as the solvent, respectively (Fig. S15†). In contrast, at higher temperatures, more ZnS and CdS reacted with the Zn_1–*x*_Cd_*x*_S interface which drastically increased the *E*_g_ and the thickness of the interfacial layer, and hence, decreased the photocatalytic activity. Moreover, the deteriorated microstructure and substantially decreased *S*_BET_ was another explanation for the depressed H_2_-evolution activity in the samples prepared at higher temperatures. The photocatalytic H_2_-production activity of ZC30-500 was considerably higher than the other CdS-based photocatalysts (Table S1†),^2a,5d,6b,7,16a,24^ indicating the extremely important role of the mediated interfacial state in improving the charge-separation efficiency and photocatalytic activity. The result of the comparison between the photocatalytic stabilities of pristine ZC30 and ZC30-500 measured without renewing the sacrificial solution is presented in Fig. 3c. The pristine ZC30 exhibited a relatively good stability of 20 h because of the intimate heterojunction, efficient spatial charge separation and suitable band-structure matching.^12*a*^ Interestingly, ZC30-500 exhibited a considerably higher H_2_-production rate and longer photocatalytic lifetime over 60 h in comparison to ZC30 because of its faster interfacial electron transfer as confirmed by its much smaller semicircle in the middle-frequency region (Fig. S13†). This result was attributed to the further improvement of interface state *via* the post-annealing process, thereby confirming the hypothesis that the improved interface state could simultaneously enhance photocatalytic activity and stability. Neither the microstructure nor the XRD patterns of ZC30-500 exhibited distinct variations after 60 h of photocatalytic reaction (Fig. S16†), further confirming its excellent photocatalytic stability.

The mechanism for the separation and transport of photon-generated charge carriers in the heterostructure is shown in Fig. 3d. Photo-generated holes from the valence band (VB) of CdS transferred to the defect states of the interfacial Zn_1–*x*_Cd_*x*_S layer, which then transferred into the acceptor states of ZnS because of the lower acceptor states (V_Zn_, I_s_) in Zn_1–*x*_Cd_*x*_S than those in ZnS. The defect states related acceptor levels in ZnS and Zn_1–*x*_Cd_*x*_S were confirmed by the photoluminescence (PL) spectra (Fig. S17†). All of the heterostructure samples and the solid solution samples prepared *via* a thermolysis method^16*a*^ with different amounts of Cd^2+^ showed a similar peak position of the emission derived from different defect states (such as V_Zn_) when compared to that in ZnS. These results indicated the constant energy level of the defect states in ZnS, heterostructure and solid solution samples with respect to their CB edge. These results were consistent with previous publications,^5d,15^ which reported that the peak position of the emission derived from the defect states (such as V_Zn_) in Zn_1–*x*_Cd_*x*_S is similar to that in ZnS and doesn't change with the variation of *E*_g_s derived from the different amounts of Cu^2+^ dopant. Consequently, the semiconductor with a lower CB edge possesses a lower defect (such as I_s_ and V_Zn_) related acceptor level. Meanwhile, the photo-excited electrons from the interfacial Zn_1–*x*_Cd_*x*_S layer transferred to the CB of the CdS QDs, which was located on the outside of the heterostructure. As a result, the excellent photocatalytic activity and stability in the ZnS/Zn_1–*x*_Cd_*x*_S/CdS heterostructure may have been a consequence of the combined effects of the following three factors: (i) the mediated Zn_1–*x*_Cd_*x*_S layer exhibited tunable parameters and lower acceptor states (such as V_Zn_) when compared to ZnS, which dramatically improved the matching between ZnS and CdS, and the separation efficiency of the photo-generated charge carriers; (ii) the post-annealing process drastically improved the interface state and the crystallinity of the ZnS/Zn_1–*x*_Cd_*x*_S/CdS heterostructure and enhanced the charge separation and transport efficiency; and (iii) the large *S*_BET_ of 105 m^2^ g^–1^ and interconnected 3D network in the mesoporous heterojunction drastically increased the number of the reaction sites.

## Conclusions

In summary, for the first time, we designed and successfully fabricated a ZnS/CdS 3D mesoporous heterostructure with a mediating Zn_1–*x*_Cd_*x*_S interface that serves as a charge carrier transport channel. ZnS(en)_0.5_ functions as both a template for the fabrication of a highly porous 3D interconnected network and as the Zn and S sources for the *in situ* formation of a stably embedded Zn_1–*x*_Cd_*x*_S interfacial layer. Furthermore, the H_2_-production rate and the stability of the heterostructure are simultaneously improved by the careful modification of the interface state *via* a simple post-annealing method. The sample prepared with the optimal parameters exhibits an excellent H_2_-production rate of 106.5 mmol h^–1^ g^–1^ under visible light, which is 152 and 966 times higher than CdS prepared using ethylenediamine and deionized water as the solvent, respectively, and is considerable higher than other CdS-based photocatalysts. Moreover, this heterostructure shows excellent photocatalytic stability over 60 h. This work not only proposed a new strategy for fabricating a water-splitting catalyst with high photocatalytic activity *via* modulating the interface state but also provides a new general route to improve the photocatalytic activity and stability of heterostructures for the effective utilization of solar energy.
