# Supplementary material for: Self-assembly of a mesoporous ZnS/mediating interface/CdS heterostructure with enhanced visible-light hydrogen-production activity and excellent stability
Source: Chem Sci. 2015 Jun 18;6(9):5263–8. doi: 10.1039/c5sc01586c (PMC5500944; doi:10.1039/c5sc01586c)
Supplement: Supplementary file 1 [file SC-006-C5SC01586C-s001.pdf]

Electronic Supplementary Material (ESI) for Chemical Science.
This journal is © The Royal Society of Chemistry 2015

Supporting Information

Self-assembly of a mesoporous ZnS/mediating interface/CdS heterostructure with enhanced visible-light hydrogen-production activity and excellent stability

Kui Li,[‡a] Rong Chen,[‡a] Shun-Li Li,[a] Min Han,[a] Shuai-Lei Xie,[a] Jian-Chun Bao,*[a] Zhi-Hui Dai[a] and Ya-Qian Lan*[ab]

* Dr. K. Li, Miss R. Chen, Prof. S.-L. Li, Prof. M. Han, Mr. S.-L Xie, Prof. Z.-H Dai, Prof. J.-C Bao, Prof. Y.-Q. Lan

[a] *Jiangsu Key Laboratory of Biofunctional Materials, School of Chemistry and Materials Science, Nanjing Normal University, Nanjing 210023, P. R. China. Email: yqlan@njnu.edu.cn; baojianchun@njnu.edu.cn.*
[b] *State Key Laboratory of Coordination Chemistry, School of Chemistry and Chemical Engineering, Nanjing University, Nanjing 210093, P. R. China.*

[‡] These authors contributed to this work equally.
E-mail: yqlan@njnu.edu.cn; baojianchun@njnu.edu.cn.

1. Experimental Section:

1.1 Chemicals: $Cd(NO_3)_2{\cdot}4H_2O$, $ZnCl_2$, thiourea and ethylenediamine (Tokyo Chemical Industry Co., Ltd) are analytical grade and used as received without further purification.

1.2 Sample preparation.

1.2.1 Synthesis of ZnS-ethylenediamine inorganic-organic ($ZnS(en)_{0.5}$) hybrid nanosheet: The $ZnS(en)_{0.5}$ was prepared using zinc chloride ($ZnCl_2$), thiourea as precursors and ethylenediamine (EDA) as solvent. $ZnS(en)_{0.5}$ inorganic-organic hybrid nanosheet was first prepared with the modified method according to the literation.[1] In a typical process, 1.5 mmol $ZnCl_2$ and 3.0 mmol thiourea were dissolved in 30 mL pure ethylenediamine under ultrasound till all the reagents were dissolved. The solution was then transfer into 50 mL teflon-lined autoclave and maintained 180 ^{o}C for 21 h. The final white products were rinsed three times with distilled water and ethanol respectively, and dried at 60 ^{o}C for overnight in vacuum oven to evaporate the solvent ethanol. The obtained $ZnS(en)_{0.5}$ was used as precursor for the fabrication of the heterostructure catalysts.

1.2.2 Synthesis of the $ZnS/Zn_{1-x}Cd_xS/CdS$ heterojunction derived from $ZnS(en)_{0.5}$: 30 mg $ZnS(en)_{0.5}$ was dissolved in 10 mL deionized water under ultrasound for a few minutes. Different contents of $Cd(NO_3)_2{\cdot}4H_2O$ was dissolved in deionized water (2.8 mL) and then drop into the aforementioned solution quickly under mild stirring. After several minutes, the obtained solution was transferred into 15 mL autoclave and maintained 140 ^{o}C for 12 h. The final products with different concentrations of Cd^{2+} were respectively rinsed with distilled deionized water and ethanol for three times, and dried at 60 ^{o}C overnight in the vacuum oven to evaporate the solvent ethanol.

1.2.2 Synthesis of the $ZnS/Zn_{1-x}Cd_xS/CdS$ heterojunction with Cd /Zn equaling to 150 at% derived from $ZnS(en)_{0.5}$: 30 mg $ZnS(en)_{0.5}$ was dissolved in 10 mL deionized water under ultrasound for a few minutes. Suitable contents of $Cd(NO_3)_2{\cdot}4H_2O$ (with Cd/Zn = 150 at%) was dissolved in deionized water and then drop into the aforementioned solution quickly under mild stirring. After several minutes, the obtained solution was transferred into 15 mL autoclave and maintained at 140 ^{o}C for 12 h. The final products were rinsed with distilled deionized water and ethanol for three times, respectively, and dried at 60 ^{o}C overnight in the vacuum oven to evaporate the solvent ethanol.

1.2.3 Synthesis of CdS(en)$_{0.5}$, mesoporous CdS (M-CdS), CdS prepared with hydrothermal method (H-CdS) and ZnS+CdS: Considering the difficulty in fabricating pure CdS with the cation exchange method, the CdS(en)$_{0.5}$ was first prepared using the same process as that of ZnS(en)$_{0.5}$. Typically, 1.5 mmol $Cd(NO_3)_2{\cdot}4H_2O$ and 3.0 mmol thiourea were dissolved in 30 mL pure ethylenediamine under ultrasound till all the reagents were dissolved. The solution was then transfer into 50 mL teflon-lined autoclave and maintained 180 ^oC for 21 h. The product was rinsed three times with distilled water and ethanol respectively, and dried at 60 ^oC for overnight in vacuum oven to evaporate the solvent ethanol.

M-CdS was prepared with further hydrothermal method. Typically, 30 mg CdS(en)$_{0.5}$ was dissolved in 12.8 mL deionized water under ultrasound for a few minutes. After several minutes, the obtained solution was transferred into 15 mL autoclave and maintained 140 ^oC for 12 h.

H-CdS was prepared with the same parameters to that of ZnS(en)$_{0.5}$ with only difference in that the solvent is deionized water.

ZnS+CdS: The CdS and ZnS (i.e. ZC0) derived from CdS(en)$_{0.5}$ and ZnS(en)$_{0.5}$ repectively were mixed by mechanical stirring with nominal Cd/Zn ratio of 30 at% in 100 ml deionized water, and then centrifugated and dried in the vacuum oven.

1.2.4 Post-annealing process of ZnS/Zn$_{1-x}$Cd$_x$S/CdS heterojunction: The optimized heterojunction loaded 30 at% Cd (ZC30) was post-annealed at 450 ^oC , 500 ^oC, 550 ^oC, 650 ^oC for 1 h with ramping rate of 5 ^oC/min in nitrogen atmosphere.

1.2.5 Inhibited formation of interface via adoption Na$_2$S in ZC30: To qualitatively confirm the extremely important role of interfacial layer on the H_2-procuction activity, the sulfur source (Na_2S) was added with Cd^{2+} to inhibit the formation of $Zn_{1-x}Cd_xS$ interface layer. Typically, 30 mg ZnS(en)$_{0.5}$ was dissolved in 10 mL deionized water under ultrasound for a few minutes. Suitable contents of $Cd(NO_3)_2{\cdot}4H_2O$ (with Cd/Zn = 30 at%) and suitable Na_2S (S/Cd=10, 50, 100 at%) were dissolved in deionized water and then drop into the aforementioned solution quickly under mild stirring. After several minutes, the obtained solution was transferred into 15 mL autoclave and maintained at 140 ^oC for 12 h. The final products were rinsed with distilled deionized water and ethanol for three times, respectively, and dried at 60 ^oC overnight in the vacuum oven to evaporate the solvent ethanol.

2. Characterization.

The powder X-ray diffraction (XRD) patterns were recorded on a D/max 2500 VL/PC diffractometer (Japan) equipped with graphite monochromatized Cu Kα radiation (λ = 1.54060 Å). Corresponding work voltage and current is 40 kV and 100 mA, respectively. The transmission electron microscopy (TEM) and high-resolution TEM (HRTEM) images were recorded on JEOL-2100F apparatus at an accelerating voltage of 200 kV. Surface morphologies of the carbon materials were examined by a scanning electron microscope (SEM, JSM-7600F) at an acceleration voltage of 10 kV. The energy-dispersive X-ray spectroscopy (EDX) was taken on JSM-5160LV-Vantage typed energy spectrometer. UV−visible diffused reflectance spectra was recorded using a Cary 5000 UV-Vis spectrometer (Viarian, USA) with $BaSO_4$ as a reflectance standard. The Brunauer−Emmett−Teller (BET) specific surface area (S_{BET}) of the heterojunction samples analyzed by nitrogen adsorption and water vapor adsorption is investigated by an Autosorb-iQ adsorption apparatus (Quantachrome instruments, USA). All of the prepared samples were degassed at 90 °C for 3 hours prior to nitrogen adsorption measurements. The BET surface area was determined by a multipoint BET method using adsorption data in the relative pressure (P/P0) range of 0.05− 0.3. A desorption isotherm was used to determine the pore size distribution via the Barret−Joyner−Halender (BJH) method, assuming a cylindrical pore model. The nitrogen adsorption volume at a relative pressure (P/P0) of 0.972 was used to determine the pore volume and average pore size. Electrochemical impedance spectra (EIS) measurements were carried out in three-electrode system and recorded over a frequency range of 0.005-10^5 Hz with ac amplitude of 10 mV at 0.5 V in dark using EC-lab (SP-150, VMP3-based instruments, France) under a surface power density of about 0.1 mW/cm^2. Na_2S (0.1 M) and Na_2SO_3 (0.02 M) mixture solution was used as the supporting electrolyte. Fourier transform infrared spectroscopy (FT-IR) spectra was recorded on an infrared spectrometer (Tensor 27, Bruker, German). The photoluminescence spectra was performed on a Molecular Fluorescence Spectrometer (Cary Eclipse, Varian Associates, America).

3. Photocatalytic Hydrogen Production.

The photocatalytic H_2 production experiments were performed in a 100 mL Pyrex flask at ambient temperature and atmospheric pressure, and the openings of the flask were sealed with

silicone rubber septum. A 300 W xenon arc lamp through a UV-cutoff filter with a wavelength range of 420−800 nm, which was positioned 13 cm away from the reaction solution, was used as a visible light source to trigger the photocatalytic reaction. The focused intensity on the flask was about 200 mW·cm^{-2}, which was measured by a FZ-A visible-light radiometer (made in the photoelectric instrument factory of Beijing Normal University, China). In a typical photocatalytic H_2-production experiment, 5 mg of the prepared ZnS/$Zn_{1-x}Cd_xS$/CdS photocatalyst was suspended in 100 mL of mixed aqueous solution containing Na_2S (0.35 M) and Na_2SO_3 (0.25 M). Before irradiation, the system was vacuumed for 5 min via the vacuum pump to completely remove the dissolved oxygen and ensure the reactor was in an anaerobic condition. As shown in Fig. S1, a continuous magnetic stirrer was applied at the bottom of the reactor to keep the photocatalyst particles in suspension during the experiments. H_2 content was analyzed by gas chromatography (GC-7900, CEAULight, China) (Fig. S0). All glassware was carefully rinsed with DI water prior to use.

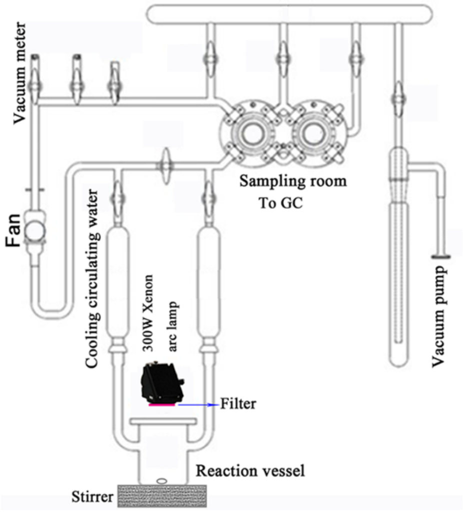

Scheme S1. The schematic diagram of the testing system. The sampling room is connected to the gas chromatography (GC-7900, CEAULight, China) with nitrogen as a carrier gas.

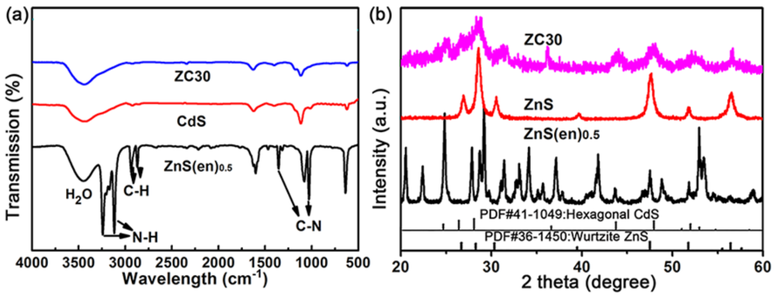

Fig. S1. The (a) FT-IR spectra and (b) XRD patterns of $ZnS(en)_{0.5}$, ZnS, ZC30, and CdS. Both the infrared absorption spectroscopy and XRD patterns confirmed that EDA is totally removed in all the samples after the hydrothermal process.

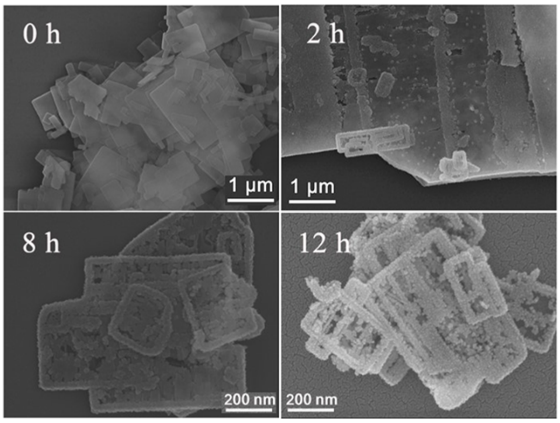

Fig. S2. The time dependence of SEM images in the heterostructure sample with 10 at% Cd. The ZnS–ethylenediamine inorganic–organic hybrid nanosheets were sculptured by Cd^{2+} layer by layer into rectangle nanoframe after the hydrothermal process at 140 °C for 12 h.

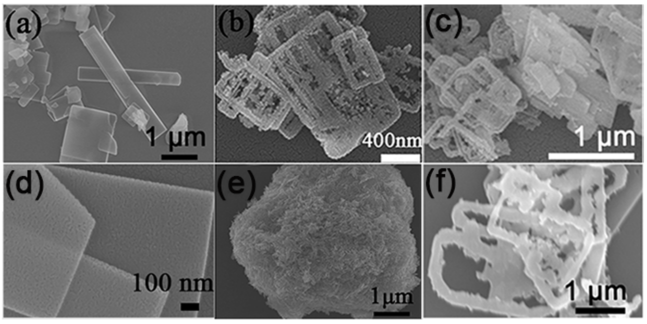

Fig. S3. SEM images of the (a) $ZnS(en)_{0.5}$ nanosheets, (b)-(d) the heterostructure samples with 10, 30 and 75 at% Cd, (e) CdS derived from CdS–ethylenediamine and (f) ZC30 post-annealed at 650 °C. The ZnS–ethylenediamine (EDA) inorganic–organic hybrid nanosheets were first sculptured by Cd^{2+} cations layer by layer into rectangle nanoframe and then mesoporous nanosheets in the samples with 75 at% Cd. While the CdS derived from the CdS–EDA shows mesoporous microstructure. In contrast to the pristine ZC30, ZC30 post-annealed at 650 °C presents a considerably denser and destructed microstructure.

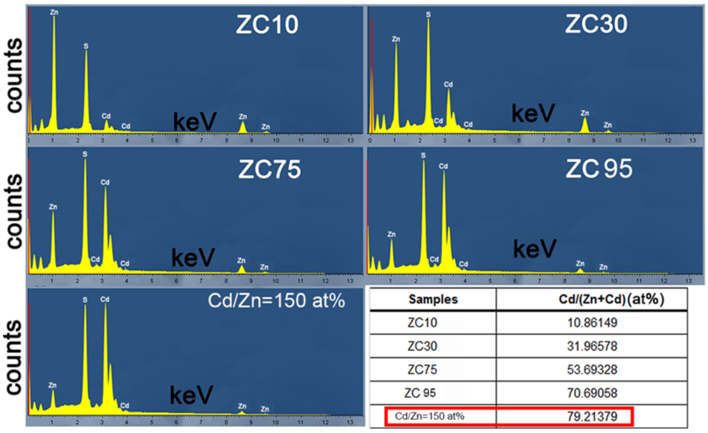

Samples	Cd/(Zn+Cd)(at%)
ZC10	10.86149
ZC30	31.96578
ZC75	53.69328
ZC 95	70.69058
Cd/Zn=150 at%	79.21379

Fig. S4. The electron dispersive X-ray (EDX) spectrum for the semiconductor heterostructure with different amount of Cd. It can be observed from the table (the ratio of Cd/Zn recorded by EDX) that the ratio from the EDX is much smaller than that of the setting value. Especially, even with Cd/Zn = 150%, the Zn can not be totally replaced by the Cd and the ratio of Cd/Zn is only 79.2 at%, indicating that the interface as well as CdS layer may inhibit the further cation exchange of ZnS.

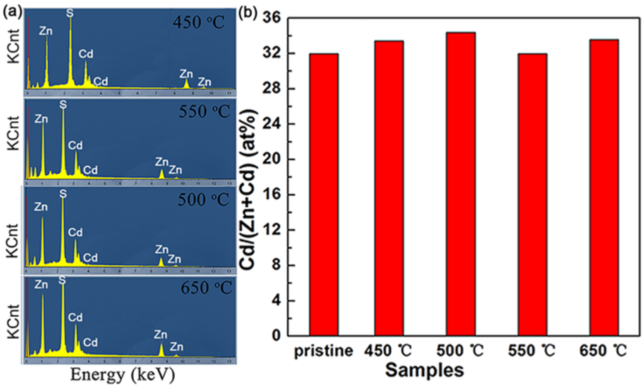

Fig. S5. The (a) electron dispersive X-ray (EDX) spectrum and (b) the ratio of Cd/Zn of the pristine ZC30 (with the ratio of Cd/Zn equaling to 30 at%) post-annealed at different temperatures. The post-annealing process shows little effect on the composition (ratio of Cd/Zn).

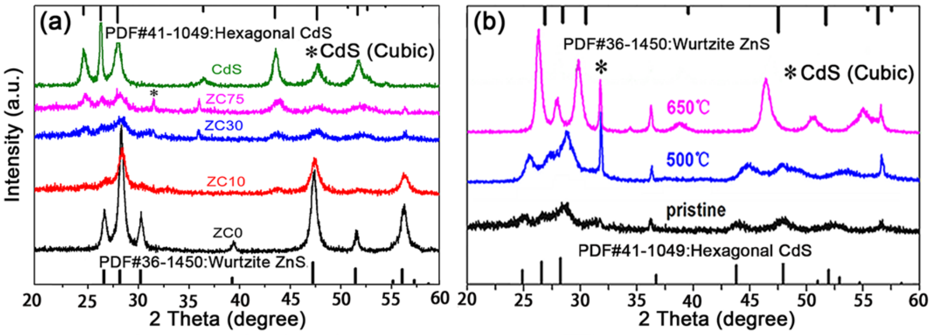

Fig. S6. The X-ray diffraction patterns of (a) heterojunction with different amounts of Cd (ZC0 to ZC75 represent the sample with the ratio of Cd/Zn equaling to 0 to 75 at% respectively) and (b)

ZC30 post-annealed at different temperatures. It can be observed that the wurtzite ZnS phase gets weaker with the increasing hexagonal and cubic phase of CdS with the corporation of Cd^{2+} because of the limited S source. However, the characteristic peak of ZnS doesn't disappear because of the larger radius of Cd^{2+} (0.97 Å) than that of the Zn^{2+} (0.74 Å). The post-annealing process dramatically improved the level of crystallization in ZC30 without the formation of any extra phases. The peak corresponding to $Zn_{1-x}Cd_xS$ shifted to higher 2-theta values with increasing temperature, indicating the decreased content of Cd in $Zn_{1-x}Cd_xS$ because of the reaction between ZnS and the interfacial layer at high temperatures.

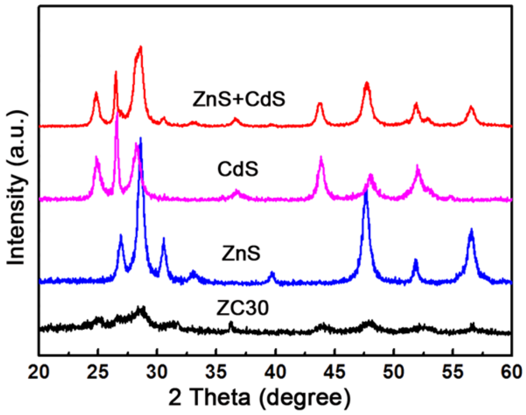

Fig. S7. The comparison results of the X-ray diffraction (XRD) patterns of the ZC30 to the ZnS and CdS mixture. The ZC30 shows the similar phase structure to that of the ZnS and CdS mechanical mixture with the only difference in that the ZC30 shows much lower level of crystallization and a little peak position shift, which further confirms the presence of the $Zn_{1-x}Cd_xS$ and ZnS phases.

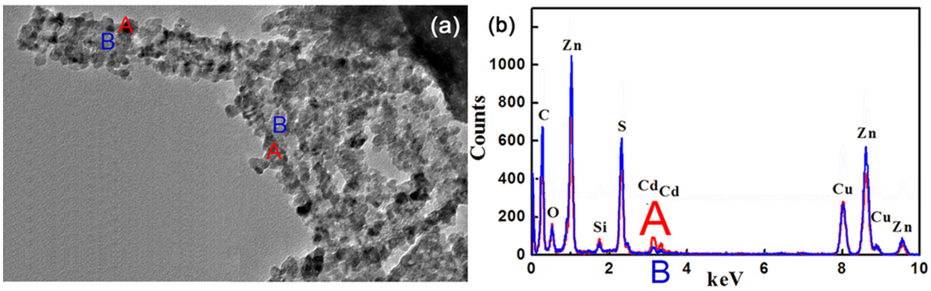

Fig. S8. (a) HRTEM and (b) EDX images at the different position of the nanoframe in ZC30. The concentration of Cd in the edge of the nanoframe is much larger than that in the center, indicating the as prepared samples a quasi-core-shell structure, which is consistent to the element mapping result.

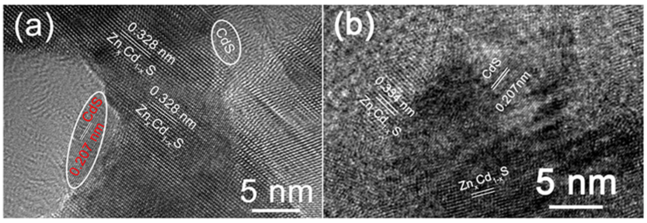

Fig. S9. HRTEM of the sample loaded 30 at% Cd (ZC30) post annealed at (a) 500 °C and (b) 650 °C. Compared with the pristine ZC30, the CdS nanoparticle in the sample post-annealed at 500 °C shows better crystallization (clear lattice fringe) while decreasing nanoparticle size. For the sample post annealed at 650 °C, CdS nanoparticle can still be observed in the surface, indicating that the interface layer can dramatically remain the heterostructure.

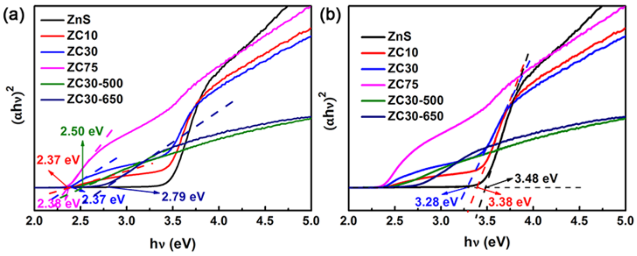

Fig. S10. The $(\alpha h\nu)^2$ versus $h\nu$ curve in the (a) visible and (b) UV region of the samples with different Cd content and ZC30 post annealed at different temperatures. The band structures of these samples in the visible and UV region is calculated by the Kubelka−Munk (KM) method according to the following equation:

$$\alpha h\nu = A(h\nu - E_g)^{1/2}$$

where α is the absorption coefficient, hν is the photon energy, Eg is the direct band gap, and A is a constant.

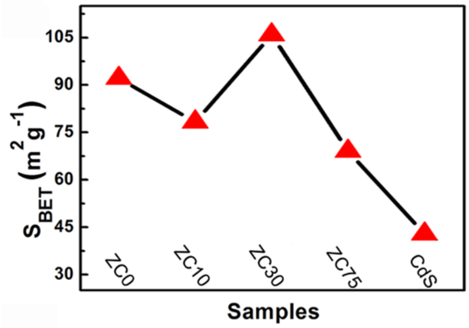

Fig. S11. The specific surface area (S_{BET}) of the heterostructure samples with different amount of Cd. The S_{BET} of ZnS (ZC0) decreases with increasing ratio of Cd/Zn to 10 at% (ZC10), while increases dramatically to ZC30. Further increasing the amount of Cd decreases the S_{BET}.

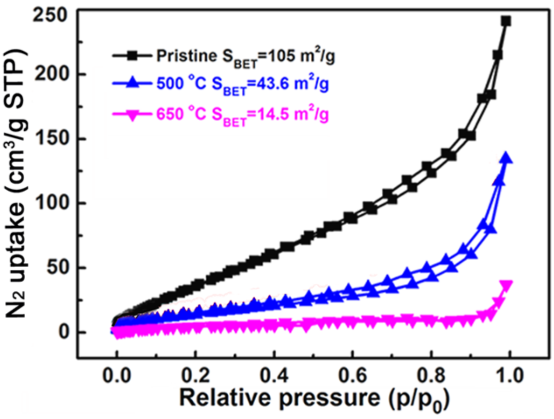

Fig. S12. Effect of post-annealing temperature on nitrogen adsorption/desorption isotherms and specific surface area. Fortunately, both N_2 adsorption and specific surface area of the heterostructure don't decrease dramatically in ZC30-500 because of its well-maintained microstructure. However, post-annealing process at 650 °C seriously decreases the specific surface area to 14.5 m^2/g stemming from destructed microstructure in ZC30-650.

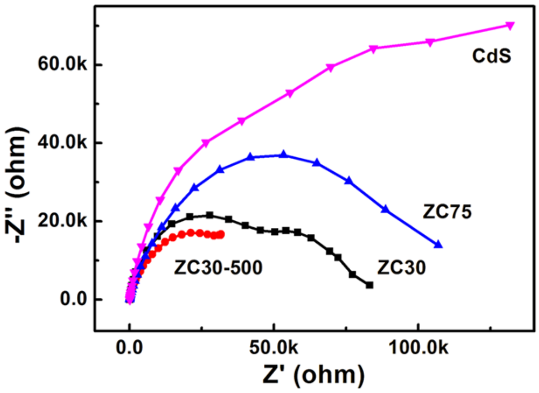

Fig. S13. Nyquist plots of ZC30-500, ZC30, ZC75 and CdS in 0.1 M Na_2S + 0.02 M Na_2SO_3 aqueous solution under a surface power density of about 0.1 mW/cm^2. Electrochemical impedance spectra (EIS) analysis is a powerful method to investigate charge transfer process occurring in the three-electrode system and the EIS. The intermediate-frequency response is associated with the electron transport and transfer at the semiconductor sample/electrode interface. The heterojunction with 30 at% Cd^{2+} (ZC30) shows much smaller semicircle in the middle-frequency region compared with that of ZC75 and CdS, indicating its faster interfacial electron transfer stemming from the well modulated interface layer. Consequently, the interface can act as an electron collector and transporter in the heterojunction and thus significantly enhance the photocatalytic H_2-production activity. Moreover, the ZC30-500 shows the smallest semicircle in the middle-frequency region among these samples, indicating the extremely important role of high level of crystallization in improving the separation of charge carriers and H_2-production activity.

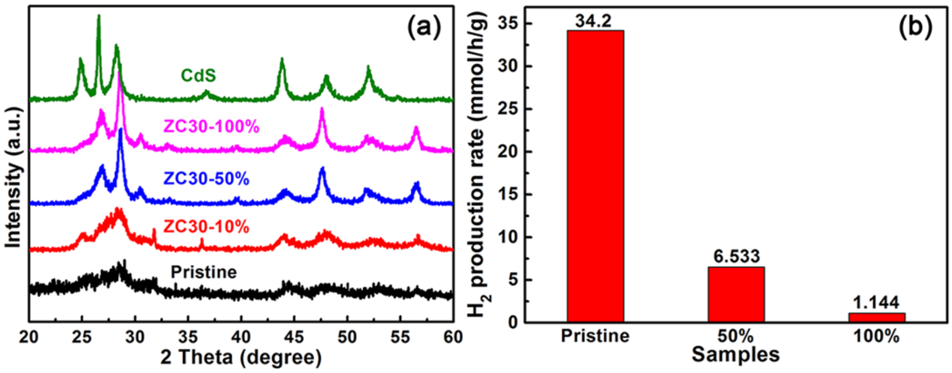

Fig. S14. Effect of the sulfur source (Na_2S) amount on the (a) XRD and (b) H_2-production activity of the samples with 30 at% Cd^{2+} (ZC30). Both the intensity of CdS and ZnS phase get stronger with increasing the amount of sulfur source. Correspondingly, the H_2-production activity decreases dramatically because of the inhibited formation of $Zn_{1-x}Cd_xS$ interface.

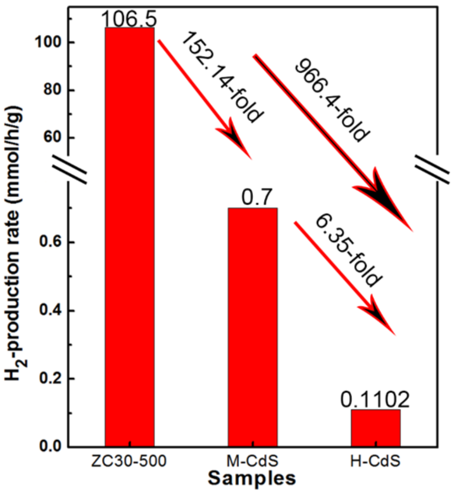

Fig. S15. Comparison of the visible light photocatalytic H_2-production activity of ZC30 post-annealed at 500 °C (ZC30-500), the mesoporous CdS derived from $CdS(en)_{0.5}$ (M-CdS) and the CdS prepared with hydrothermal method (H-CdS). The H_2-evolution rate of M-CdS exceeds that of H-CdS by 6 times because of its high porous microstructure. The ZC30 post-annealed at 500 °C shows the highest of 106 mmol/h/g among the various CdS-based photocatalysts, exceeding that of M-CdS and H-CdS by more than 152 and 966 times respectively.

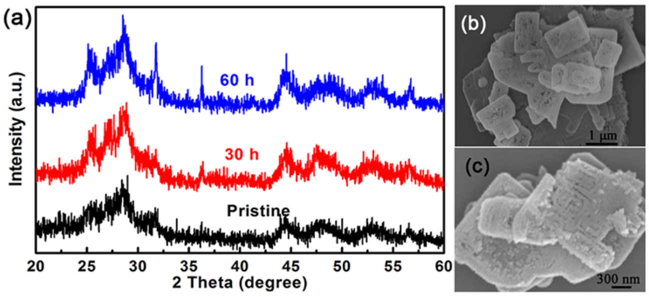

Fig. S16. (a) Effect of reaction time on the XRD results of photocatalytic sample. Comparison of the SEM microstructure morphology of (b) ZC30-500 and (c) the samples irradiated after 60 h.

Both microstructure and phases in ZC30-500 don't show dramatically variation after photocatalytic reaction of 60 h, further confirming the excellent lifetime of this sample.

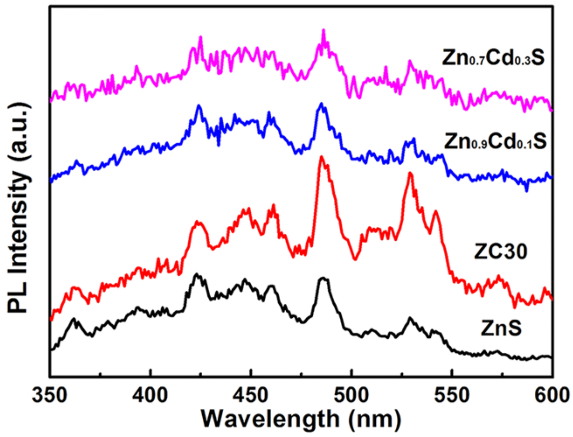

Fig. S17. Room temperature PL excitation spectra of the heterostructure samples and the samples prepared via thermolysis method. The relative defect (V_{Zn}) related acceptor levels in ZnS and $Zn_{1-x}Cd_xS$ were confirmed by the photoluminescence (PL) spectra (Fig. S17). All the heterostructure samples and the solid solution prepared via thermolysis method (*ACS Catal.*, **2013**, *3*, 882−889) with different amount of Cd^{2+} show the similar peak position (such as the peak at around 480 nm from V_{Zn}) of the emission derived from different defect states to that in ZnS, which indicates the constant energy level of the defect states in these samples with respect to their conduction band edge. Consequently, the semiconductor with lower conduction band edge possesses lower defect states (such as I_s and V_{Zn}) related local acceptor level. These results confirmed that the potogenerated holes can transform from VB of CdS to V_{zn} of ZnS with higher efficiency through the interfacial transport channel ($Zn_{1-x}Cd_xS$).

Table S1. Comparison results of H_2-production rate in the CdS based photocatalysts via water splitting.

Photocatalyst	H_2 evolution			Ref.(year)
	Activity (mmol/h/g)	Improving multiple[a]	Stability (h)	
ZC30-500	**106.5**	**152**	>60	**This work**
ZC30	**34.2**	**48**	≈20	**This work**
CdS/ZnS	**0.792**	**56**	>60	[2] (2014)
CdS/MoS_2	**0.59**	**36**	N/A	[3] (2008)
$Zn_{1-x}Cd_xS$	**7.42**	**24**	N/A	[4] (2013)
MoS_2–rGO/CdS	**23.2**	**12.39**	>12	[5](2014)
CdS/Cd	**11.687**	**7.012**	N/A	[6] (2014)
CdS/RGO	**56**	**4.87**	N/A	[7] (2011)
1D-$Cd_{0.8}Zn_{0.2}S$	**N/A**	**3.01**	<12	[8](2015)
CdS/g-C_3N_4	**4.152**	**2.075**	N/A	[9] (2013)

CdS/ZnS	**N/A**	**≈2**	N/A	[10] (2013)

[a]Improving multiple=H_2-production rate of the optimal catalyst/H_2-production rate of CdS.

References:

[1] J. Zhang, J. Yu, Y. Zhang, Q. Li and J. R. Gong, *Nano Lett.*, **2011**, *11*, 4774-4779.

[2] Y. P. Xie, Z. B. Yu, G. Liu, X. L. Ma and H.-M. Cheng, *Energy Environ. Sci.*, **2014**, *7*, 1895-1901.

[3] X. Zong, H. Yan, G. Wu, G. Ma, F. Wen, L. Wang and C. Li, *J. Am. Chem. Soc.*, **2008**, *130*, 7176-7177.

[4] Q. Li, H. Meng, P. Zhou, Y. Zheng, J. Wang, J. Yu and J. Gong, *ACS Catal.*, **2013**, *3*, 882−889.

[5] M. Liu, F. Li, Z. Sun, L. Ma, L. Xu and Y. Wang, *Chem. Comm.*, **2014**, *50*, 11004-11007.

[6] Q. Wang, J. Li, Y. Bai, J. Lian, H. Huang, Z. Li and Z. Lei and W. Shangguan, *Green Chem.*, **2014**, *16*, 2728-2735.

[7] Q. Li, B. Guo, J. Yu, J. Ran, B. Zhang, H. Yan and J. R. Gong, *J. Am. Chem. Soc.*, **2011**, *133*, 10878-10884.

[8] Z. Han, G. Chen, C. Li, Y. Yu and Y. Zhou, *J. Mater. Chem. A*, **2015**, *3*, 1696-1702.

[9] J. Zhang, Y. Wang, J. Jin, J. Zhang, Z. Lin, F. Huang and J. Yu, *ACS Appl. Mater. Interfaces*, **2013**, *5*, 10317-10324.

[10] L. Huang, X. Wang, J. Yang, G. Liu, J. Han and C. Li, *J. Phys. Chem. C*, **2013**, *117*, 11584-11591.
